# Supplementary material for: Unveiling Species Diversity Within Early-Diverging Fungi from China VII: Seven New Species of Cunninghamella (Mucoromycota)
Source: J Fungi (Basel). 2025 May 29;11(6):417. doi: 10.3390/jof11060417 (PMC12194472; doi:10.3390/jof11060417)
Supplement: Supplementary file 1 [file jof-11-00417-s001.zip › Table S1.pdf]

Table S1. GenBank accession numbers of sequences used in this study.

Species	Strains	GenBank accession numbers		
		ITS	LSU	*TEF1α*
Cunninghamella antarctica	CBS 545.75*	JN205893	JN206597	KJ156492
C. arunalokei	NCCPF 890012*	NR_177485	NG153887	NA
C. arunalokei	IL3459	MN431159	MN431158	NA
C. arrhiza	CGMCC 3.16111*	OL678142	PQ399916	NA
C. arrhiza	XY08047	OL678143	NA	NA
C. amphispora	**CGMCC 3.28652***	**PV089203**	**PV123104**	**PV200769**
C. amphispora	**XG09634-9-2**	**PV089204**	**PV123105**	**PV200770**
C. bertholletiae	CBS 693.68	AF254931	MH870924	KJ156490
C. bertholletiae	CBS 190.84	JN205878	HM849701	NA
C. binariae	NRRL 1375*	AF254935	NA	NA
C. binariae	CBS 481.66	MH858865	MH870507	KJ156495
C. bigelovii	CGMCC 8094*	KJ013403	KJ013405	KJ395944
C. blakesleeana	CBS 133.27*	NR119974	MH866397	KJ156479
C. blakesleeana	CBS 782.68	JN205869	MH870950	KJ156478
C. clavata	Cu-15	AF254942	NA	NA
C. clavata	CBS 362.95	JN205891	NA	KJ156473
C. clavata	CBS 100178	JN205890	JN206604	KJ156477
C. cinerea	**CGMCC 3.28650***	**PV089197**	**PV123098**	**PV172612**
C. cinerea	**XG09556-9-2**	**PV089198**	**PV123099**	**PV172613**
C. echinulata	CBS 156.28*	JN205895	HM849702	KJ156500
C. elegans	CBS 160.28*	AF254928	NR_154747	KJ156470
C. elegans	CBS 167.53	JN205882	HM849700	KJ156494
C. elegans	EML-RUS1-1	MF806023	MF806027	NA
C. elegans	EML-RUS1-2	MF806021	MF806028	NA
C. flava	**CGMCC 3.28651***	**PV089199**	**PV123100**	**PV200765**
C. flava	**XG09559-10-2**	**PV089200**	**PV123101**	**PV200766**
C. globospora	CGMCC 3.16020*	MW264073	MW264132	NA
C. gigacellularis	URM 7400*	NR_168760	NG_068773	NA
C. homothallica	IFO 6736	AF254941	NA	NA
C. homothallica	CBS 168.53*	JN205863	JN206605	KJ156498
C. intermedia	IMI 200623*	AF254939	NA	NA
C. intermedia	CBS 347.69	AF254940	NA	NA
C. irregularis	CGMCC 3.16113*	OL678145	PQ399918	NA
C. irregularis	XY07683	OL678147	NA	NA
C. irregularis	XY07657	OL678146	NA	NA
C. guizhouensis	GZUIFR-SX25*	MN908596	MN908599	MN912633
C. guizhouensis	GZUIFR-SX26	MN908597	MN908600	MN912634
C. guizhouensis	GZUIFR-SX27	MN908598	MN908601	MN912635
C. guttata	CGMCC 3.16112*	OL678144	PQ399917	NA

C. hainanensis	**CGMCC 3.28649***	**PV089195**	**PV123096**	**PV172610**
C. hainanensis	**XG06926-15-2**	**PV089196**	**PV123097**	**PV172611**
C. multiverticillata	Cu-137*	AF254933	NA	NA
C. nodosa	Cu-34*	AF346407	NA	NA
C. phaeospora	CBS 692.68*	JN205864	HM849697	NA
C. polymorpha	CBS 779.68	JN205874	JN206599	NA
C. polymorpha	CBS 693.68	JN205871	JN206600	NA
C. regularis	CGMCC 3.16114*	OL678148	PQ399919	NA
C. regularis	XY07510	OL678149	NA	NA
C. regularis	XY07512	OL678150	NA	NA
C. regularis	XY07516	OL678151	NA	NA
C. rhizoidea	**CGMCC 3.28654***	**PV089205**	**PV123106**	**PV222157**
C. rhizoidea	**XG09702-9-2**	**PV089206**	**PV123107**	**PV222158**
C. saisamornae	SDBR-CMU291	MG571234	MW699591	MW715865
C. saisamornae	SDBR-CMUPFCM-6	MW709394	MW699571	MW715866
C. septata	Cu-230*	AF346408	NA	NA
C. subclavata	CGMCC 3.16115*	OL678152	NA	NA
C. subclavata	XY07766	OL678153	NA	NA
C. simplex	**CGMCC 3.28653***	**PV089201**	**PV123102**	**PV200767**
C. simplex	**XG09611-12-2**	**PV089202**	**PV123103**	**PV200768**
C. verrucosa	CGMCC 3.16260*	ON262555	ON261192	NA
C. verrucosa	XY09506	ON262556	ON261193	NA
C. verticillata	CBS595.68*	AF254937	NA	NA
C. vesiculosa	CBS 989.96*	JN205897	HM849693	KJ156474
C. vesiculosa	NRRL 3009	AF254943	NA	NA
C. varians	CGMCC 3.16116*	OL678154	PQ399920	NA
C. varians	XY06999	OL678155	NA	NA
C. yunnanensis	**CGMCC 3.28655***	**PV089207**	**PV123108**	**PV222159**
C. yunnanensis	**XG10042-9-2**	**PV089208**	**PV123109**	**PV222160**
Mucor janssenii	CBS 205.68*	MH859119	MH870832	NA

Notes: New species discovered herein are shown in bold. The asterisk "*" indicates the ex-type or ex-holotype strains. The "NA" stands for "not available".
